# The Risk of Preterm Birth in Women with Three Consecutive Deliveries—The Effect of Number and Type of Prior Preterm Births

**DOI:** 10.3390/jcm9123933

**Published:** 2020-12-04

**Authors:** Liran Hiersch, Yael Pasternak, Nir Melamed, Moshe Meshulam, Reut Shashar, Eran Hadar, Amir Aviram, Yariv Yogev, Eran Ashwal

**Affiliations:** 1 Department of Obstetrics and Gynecology, Lis Hospital for Women, Sourasky Medical Center, Tel Aviv 3QJQ+3X, Israel; yarivyogev@hotmail.com (Y.Y.); eran.ashwal@gmail.com (E.A.); 2 Sackler Faculty of Medicine, Tel Aviv University, Tel Aviv 4R73+8Q, Israel; yaeli.pasternak@gmail.com (Y.P.); moshe.meshulam@mail.huji.ac.il (M.M.); reutshashar@gmail.com (R.S.); eranh42@gmail.com (E.H.); amiraviram25@gmail.com (A.A.); 3 Department of Obstetrics and Gynecology, Meir Medical Center, Kefar Sava 5VJW+W5, Israel; 4 Division of Maternal-Fetal Medicine, Department of Obstetrics and Gynecology, Sunnybrook Health Sciences Centre, University of Toronto, Toronto, ON M4N 3M5, Canada; nirmelamed2@yahoo.com; 5 Helen Schneider Hospital for Women, Rabin Medical Center, Petach Tikva 3VQ8+MP, Israel

**Keywords:** preterm birth, recurrence, indicated, spontaneous, preterm delivery

## Abstract

**Background:** We aimed to explore the association of the number, order, gestational age and type of prior PTB and the risk of preterm birth (PTB) in the third delivery in women who had three consecutive singleton deliveries. **Methods:** A retrospective cohort study of all women who had three consecutive singleton births at a single medical center over a 20-year period (1994–2013). The primary outcome was PTB (<37 weeks) in the third delivery. **Results:** 4472 women met inclusion criteria. The rate of PTB in the third delivery was 4.9%. In the adjusted analysis, the risk of PTB was 3.5% in women with no prior PTBs; 10.9% in women with prior one PTB only in the first pregnancy; 16.2% in women with prior one PTB only in the second pregnancy; and 56.5% in women with prior two PTBs. A similar trend was observed when the outcome of interest was spontaneous PTB and when the exposure was limited to prior spontaneous or indicated PTB. **Conclusions:** In women with a history of PTB, the risk of recurrent PTB in subsequent pregnancies is related to the number and order of prior PTBs. These factors should be taken into account when stratifying the risk of PTB.

## 1. Introduction

Prematurity is considered as the leading cause of neonatal morbidity and mortality [1,2,3] with approximately 11% and 2% of viable pregnancies resulting in delivery prior to 37 and 34 weeks of gestation, respectively [1,4]. Spontaneous preterm birth (PTB) is considered a final common pathway of many biological mechanisms that vary between individuals [4]. These etiologic pathways are still yet to be elucidated [4,5].

Although there are numerous etiologies for PTB [5], a history of PTB is considered one of the strongest risk factors for reoccurrence [6,7,8]. However, while there is great amount of evidence regarding the increased risk for PTB in women with a previous single PTB [9,10,11,12], there is limited information regarding the effect of more than one prior PTB on that risk, and how different combinations of prior term and preterm births can modify the risk for PTB in the third delivery [13,14,15,16]. In addition, over the last few decades, there has been an increase in the rate of indicated PTB, mainly in the late preterm (34–36 weeks of gestation) period without a clear understanding on their effects on future deliveries [1,17]. The lack of distinguishing between indicated and spontaneous PTB [13,16] and the unknown parity at the time of the index pregnancy [16] can also limit the interpretation of the results of prior studies regarding the effect of obstetrical history on future risk for PTB.

Thus, our aim was to explore the effects of the number, order, gestational age and type of prior PTB on the risk for PTB in the third delivery in a cohort of women who had three first singleton consecutive deliveries.

## 2. Material and Methods

### 2.1. Study Design and Participants

A retrospective cohort study of all women who had their first three consecutive singleton births (≥24 + 0 weeks of gestation) at a single medical center over a 20-year period (1994–2013). Women with multiple gestations, known fetal structural or chromosomal anomalies or stillbirth in either pregnancy were excluded from the analysis. In addition, pregnancies of unknown gestational age in any one of the first three deliveries or women who did not deliver all three first consecutive deliveries in our medical center were excluded as well.

### 2.2. Exposure

The primary exposure was a history of PTB < 37 weeks in the first 2 births, and was classified into four groups based on the number and order of prior PTBs: (1) No prior PTBs (Term-Term group, which served as reference); (2) One PTB only in the first pregnancy (PTB-Term group); (3) One PTB only in the second pregnancy (Term-PTB group); and (4) PTBs in both the first and second pregnancies (PTB-PTB group). Secondary exposures were spontaneous and indicated PTBs and PTB < 34 weeks.

### 2.3. Outcomes

The primary outcome was PTB < 37 weeks in the third pregnancy. Secondary outcomes were spontaneous PTB < 37 weeks and PTB < 34 weeks in the third pregnancy.

### 2.4. Data Collection

Data were extracted from the electronic medical records. The following demographic and obstetric variables were recorded: maternal age, gravidity, parity, gestational age at delivery, hypertensive disorders (gestational hypertension or preeclampsia), or small for gestational age (SGA) defined as birthweight <10th, according to local birthweight curves, adjusted for gestational age at delivery and neonatal sex [18].

Gestational age at delivery was determined by the last menstrual period and was verified by first trimester ultrasound when available. In cases of in vitro fertilization, gestational age was determined according to the date of embryo transfer.

Hypertensive disorders of pregnancy included the presence of either gestational hypertension or preeclampsia/eclampsia. Gestational hypertension was defined as new onset (≥20 weeks of gestation) systolic blood pressure (SBP) ≥140 mmHg and/or a diastolic blood pressure (DBP ≥ 90 mmHg. Preeclampsia was defined and gestational hypertension in the presence of new onset proteinuria of ≥300 mg/d or the presence of HELLP syndrome (hemolysis, elevated liver enzymes, and a low platelet count).

During the study period, three different electronic medical records were used. Notwithstanding their extraction from different resources, all relevant data were filled during and immediately following delivery by either the attending physician, registered nurse or midwife. Furthermore, the data used in the current study were primarily discrete fields and as such less prone to error. Lastly, in terms of data quality assurance, internal consistency cross-checks are performed at regular time intervals by a specified medical staff.

The Rabin Medical Center review board (IRB) approval was obtained (0290-15-RMC).

### 2.5. Data Analysis

Data analysis was performed with the SPSS Version 27.0. Armonk, NY, USA: IBM. Normality was evaluated using the Shapiro–Wilk test. Standard Chi-square test and ANOVA test were used to compare categorical and continuous variables, respectively. Multivariable logistic regression analysis was used to determine the association between PTB in the third delivery and prior deliveries. Models were adjusted for maternal age, chronic hypertension, hypertensive disorders of pregnancy (gestational hypertension or preeclampsia), small for gestational age at delivery, gestational age at delivery, and neonatal sex as potential confounders. Additionally, we further evaluated the influence of indicated versus spontaneous prior preterm births on the risk for preterm delivery in the third delivery. The models were tested for goodness-of-fit. Differences were considered significant when *p* value was less than 0.05.

## 3. Results

### 3.1. Characteristics of the Study Population

**Figure 1:**
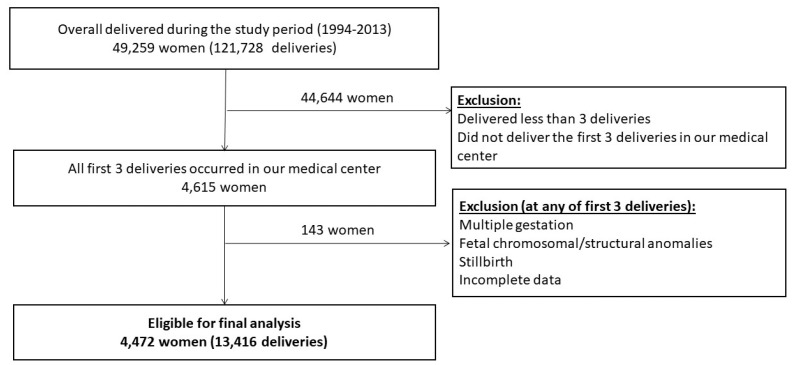
Selection of the study cohort.

During the study period, 49,259 women (121,728 deliveries) delivered in our medical center. Of them, 4615 women (13,845 deliveries) delivered their first three consecutive deliveries in our institution and 4472 of them (13,416 deliveries) met inclusion criteria (Figure 1).

Maternal mean age at the third delivery was 32.5 ± 4.2 years. In terms of gravidity, the median gravidity of our cohort at third delivery was 3 (interquartile range of 3–4). Regarding obstetrical history, amongst our study population 64.2% (*n* = 2869) had only three pregnancies at the time of their third delivery and 89.3% had no more than one additional fetal demise (<24 weeks). Of all, only 3.1% had more than two prior pregnancy losses (<24 weeks) at the time of their third delivery.

Table 1 outlines the demographic and obstetrical characteristics of the study cohort. As parity increased, the risk of hypertensive disorders (gestational hypertension and preeclampsia) significantly decreased. The overall rate of PTB at first birth was 6% and 1.2% for deliveries <37 and <34 weeks gestation, respectively. While significantly peaked in the first delivery, the rate of PTB (<37 weeks or <34 weeks) was similar between second and third deliveries (*p* = 0.824 and *p* = 0.710, respectively).

The distribution of our study population, according to the rate of PTB < 37 weeks’ gestation in the first two deliveries was as follows: 46 women (1.0%) had PTB in both the first and second deliveries (PTB-PTB group), 221 women (4.9%) had PTB in the first delivery but not in the second delivery (PTB-Term group), 167 women (3.7%) had PTB in the second delivery but not in the first delivery (Term-PTB group), and 4038 women (90.3%) had term delivery in both the first and second deliveries (Term-Term group, reference).

### 3.2. Number and Gestational Age at Prior PTB and the Risk for PTB in the Third Delivery

**Figure 2:**
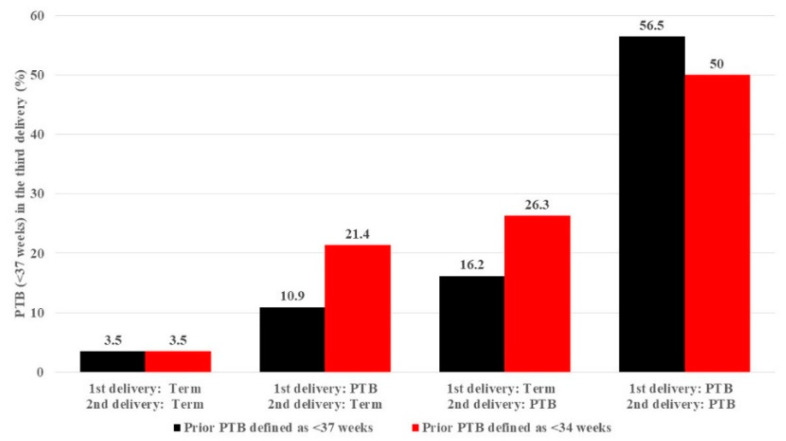
The rate of preterm birth (PTB) at the third delivery stratified by the number and gestational age of prior preterm births.

The rate of PTB (<37 weeks) in the third birth stratified by the number of and gestational age at prior preterm births is depicted in Figure 2. The rate of PTB in the third birth significantly increased as the number of prior PTBs increased. This dose-dependent pattern was shown both when the exposure was considered as prior PTB < 37 weeks of gestation (Figure 2, black columns) and also as prior PTB < 34 weeks of gestation (Figure 2, red columns) (*p* < 0.001). In multivariate logistic regression adjusted for maternal age, chronic hypertension, hypertensive disorders of pregnancy and small for gestational age status, the adjusted odds ratio (aOR) for PTB in the third birth increased significantly with a greater number of prior pregnancies complicated by PTB (Table 2). This association held true when the outcome of interest was limited to *spontaneous* PTB (<37 weeks) in the third delivery.

The rate of PTB < 37 weeks of gestation in the third delivery according to the number of prior PTB and whether prior PTB were defined as delivery <37 weeks of gestation (black bars) or <34 weeks of gestation (red bars) (Chi Square for trend <0.001 for both analyses).

**Figure 3:**
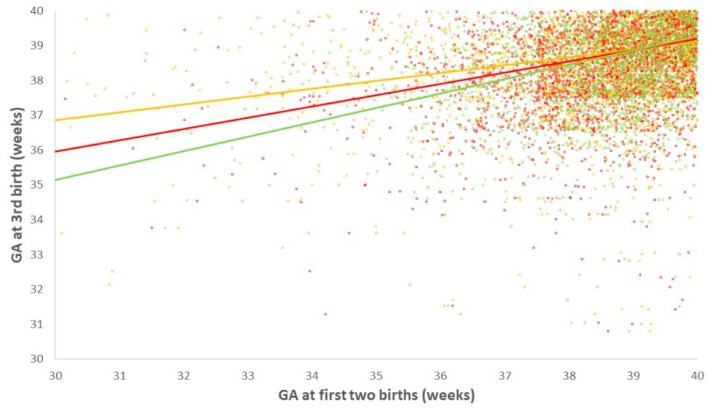
Relationship between gestational age at the third delivery and gestational age at the first two deliveries.

The timing of a single prior PTB in the first two deliveries was associated differently with the rate of PTB in the third delivery, which was more pronounced when the prior PTB was more recent to the third delivery (i.e., at the second delivery) as compared to the rate when the first PTB was followed by a term delivery (Figure 2). Similarly, gestational age at the third birth was more strongly related to gestational age at the second birth (Figure 3, red line) than the first birth (Figure 3, orange line), and was most strongly related to the mean of gestational age in both first and second births (Figure 3, green line).

This scatter plot presents the GA at the third birth in relation to GA at the first birth (orange dots), GA at the second birth (red dots), or the simple mean of GA in the first and second births (as a measure that combined information from both births). The corresponding linear regression lines are presented as well: GA[third delivery] = 0.22 × GA[first delivery] + 30.3 (orange line); GA[third delivery] = 0.32 × GA[second delivery] + 26.2 (red line); and GA[third delivery] = 0.41 × (Mean GA[first delivery] and GA[second delivery]) + 22.9 (green line). The coefficients of the 3 regression lines are significantly different from each other as determined using ANOVA (*p* < 0.05). A greater coefficient means stronger relationship with GA at the 3rd delivery. This means that GA at the 3rd delivery was more strongly related to GA at the 2nd delivery (red line) than the 1st delivery (orange) line, and was most strongly related to the mean of GA in both first and second deliveries (green line). GA, gestational age.

### 3.3. The Association of the Type of Prior PTB and the Risk for PTB in the Third Delivery

Furthermore, in order to delineate the influence of the type of prior PTB (spontaneous or indicated), we performed a multivariate logistic regression analysis controlling for maternal age, chronic hypertension, hypertensive disorders of pregnancy and small for gestational age status stratifying the cohort by the type of prior PTB (Table 3 and Table 4). Table 4 outlines the association between prior *spontaneous* PTB and the risk for spontaneous PTB in the third delivery. The risk for spontaneous PTB (<37 weeks) in the third pregnancy increased as the number of prior spontaneous PTBs increased in a dose-dependent manner. The same trend was evident when the outcome of interest was defined as PTB < 34 weeks (Table 3). Of note, 16 women were excluded from this sub-analysis due to unclear data regarding the type of prior PTB (spontaneous vs. indicated).

Lastly, we specifically evaluated the association of prior *indicated* PTB and the risk for spontaneous PTB in the third delivery (Table 4). As was observed for prior spontaneous PTBs, the risk for spontaneous PTB (<37 weeks and <34 weeks) was significantly increased with the number of prior indicated PTB.

## 4. Discussion

In the current study, we aimed to explore the association of the number, severity and type of prior preterm births and the risk of PTB in the third delivery. Our main findings were: (1) The second birth is more predictive than the first birth of preterm birth in the third birth and combining the information from both births is even more strongly related to the risk of PTB in the third birth. (2) Gestational age at prior PTB was inversely associated with the rate of PTB in the third delivery; (3) The risk of sPTB in the third delivery was increased as the number of prior spontaneous as well as indicated PTBs increased.

Not many have previously examined the effects of more than one prior PTB on the risk of PTB in the third delivery with specific consideration of the type of prior PTB [13,14,15,16]. Esplin et al. [13], reported that a previous PTB, either at the first or second delivery, increased the risk for reoccurrence in the third delivery as compared to cases with no prior PTB. However, the different combinations of preterm/term births were not analyzed separately for women with a history of only a single prior PTB. Mcmanemy et al. [16] found in a cohort of women with three consecutive deliveries a high rate of PTB in the third delivery for women with a history of PTB, ranging from 13–42%. In concordance with our results, the risk was related to the number of prior PTBs. It was also associated with the order of preterm/term births for those with only one prior PTB. Of note, their cohort was not limited to the first three deliveries of each woman. As a consequence, some women might have had more prior deliveries than those accounted for in the study, which in turn, might have influenced the risk for prematurity in the index pregnancy. Recently, in a study assessing the risk of PTB in the third delivery based on obstetrical history, Kamphuis et al. [14] showed that the outcome of the second pregnancy was more predictive of PTB in the third pregnancy than the outcome of the first pregnancy. Moreover, sPTB in the second delivery was the most predictive factor for sPTB in the third pregnancy (aOR = 8.2). Our results also indicate that the second birth is more strongly related with gestational age at the third birth. Moreover, information from both prior births is important and can be combined for better risk stratification.

Interestingly, we found that not only a history of spontaneous PTB but also indicated PTB is a major risk factor for future spontaneous PTBs. Although the mechanism of the effect of iatrogenic PTB on a future spontaneous process is unclear, several potential explanations for this finding can be offered. Maternal serum C-reactive protein (CRP) levels, reflecting maternal inflammatory response, were found to be increased in the early postpartum period in women with indicated preterm birth even as compared to those who had spontaneous preterm labor [19]. Moreover, maternal cortisol levels in women with PTB were increased in similar magnitude in those with spontaneous and indicated PTB [20], suggesting that maternal stress response is higher in preterm birth unrelated to the underlining cause. Since those variables are not routinely used in the assessment of women with PTB we are unable to confirm this hypothesis in our cohort. Finally, while term induction of labor was not shown to increase the risk for sPTB in subsequent pregnancy [21], induction of labor in the preterm period was reported as a risk factor for future spontaneous delivery [9,15]. These observations suggest that indicated PTB can also alter maternal physiology. Some of these pathological processes have been described as potential etiologies resulting in the preterm parturition syndrome [5], which may explain our findings regarding the increased risk of future spontaneous PTB in women with a history of indicated preterm births.

Current guidelines focus on measures, such as, progesterone or cervical cerclage for the prevention of PTB in women with prior sPTB [22,23]. However, the results of the current study and others [14,15] suggest that indicated preterm birth comprise a high risk of sPTB as well. Yet, preventive measures for woman with prior indicated PTB are mainly aimed to prevent reoccurrence of placenta-related complications, such as preeclampsia and fetal growth restriction [24,25,26,27] and are less common in the context of risk reduction of spontaneous PTB.

Our study withholds some strengths. First, the use of various exposure subgroups and outcomes addressing both the history and type of prior preterm births allowed us to better delineate the risk of PTB in third delivery. Our results can assist in counseling woman with a history of PTB regarding their potential risk of reoccurrence. In addition, the use of variables taken of our hospital datasets and manually exploring the files of those with PTB reduce the chance of reporting biases.

However, our study is not without limitations. Due to the retrospective nature of our design, potential confounders for PTB, such as ethnicity, the use medical treatments and maternal body-mass index [28,29,30] were unavailable. Since the population served by our institution is mainly of Caucasian ethnicity, caregivers who consult women from other ethnicities should interrupt our results with caution. In addition, as our registry does not include births <24 weeks, some women with late miscarriages or PTB < 24 weeks may have misclassified. Yet, most of our cohort (~65%) had no other pregnancies that ended <24 weeks and the vast majority (~90%) had no more than one additional pregnancy. Finally, our study spreads over a period of 20 years, during which there have been various changes in prevention measures of PTB, such as progesterone, cerclage or pessary [22,23,31]. Although our dataset lacks information regarding the uses of these measures, new evidence suggests that the effect of these measures for prevention of PTB may be more limited than once thought, and therefore, less likely to bias our results [32,33].

## 5. Conclusions

The risk of PTB in the third delivery increases as the number of prior pregnancies complicated by PTB increases, whether they were indicated or spontaneous preterm births. Although gestational age at the second birth is more related to that of the third birth, information from both prior births should be gathered and combined for better risk stratification. Future studies addressing preventive measures for women with prior indicated preterm birth are warranted.

## Figures and Tables

**Table 1:** Characteristics of the study cohort stratified by delivery order.

Variable	1st Delivery (*n* = 4472)	2nd Delivery (*n* = 4472)	3rd Delivery (*n* = 4472)
Maternal Age (y) *	26.4 ± 3.8	29.1 ± 3.9	32.5 ± 4.2
Maternal age > 35 y *	74 (1.7)	245 (5.5)	1513 (33.8)
Hypertensive disorders *	170 (3.8)	56 (1.3)	42 (0.9)
Gestational age at delivery (weeks) *	39.4 ± 1.7	39.0 ± 1.6	38.9 ± 1.6
PTB < 37 weeks *	269 (6.0)	214 (4.8)	217 (4.9)
Indicated *	168 (3.7)	104 (2.3)	100 (2.2)
Spontaneous	99 (2.2)	109 (2.4)	117 (2.6)
PTB < 34 weeks *	53 (1.2)	33 (0.7)	36 (0.8)
Indicated *	40 (0.9)	22 (0.5)	21 (0.5)
Spontaneous	13 (0.3)	11 (0.2)	15 (0.3)
Birthweight (g) *	3147 ± 497	3250 ± 463	3268 ± 466

Data are mean ± standard deviation or n (%). * Variables were statistically significant among groups (*p* < 0.01). PTB, preterm birth.

**Table 2:** The risk for preterm birth in the third delivery stratified by obstetrical history of preterm births.

Study Subgroups			Risk for PTB (<37 Weeks) in 3rd Delivery			Risk for Spontaneous PTB (<37 Weeks) in 3rd Delivery		
1st Delivery	2nd Delivery	*N* (%)	Rate of PTB *n* (%)	Crude OR (95% C.I)	Adjusted OR * (95% C.I)	Rate of PTB *n* (%)	Crude OR (95% C.I)	Adjusted OR * (95% C.I)
Term	Term	4038 (90.3)	140 (3.5)	reference	reference	65 (1.6)	reference	reference
Preterm	Term	221 (4.9)	24 (10.9)	3.39 (2.15–5.35)	3.08 (1.92–4.92)	11 (5.0)	3.20 (1.66–6.16)	3.11 (1.61–6.03)
Term	Preterm	167 (3.7)	27 (16.2)	5.37 (3.44–8.38)	5.64 (3.59–8.84)	20 (12.0)	8.32 (4.91–14.09)	8.58 (5.05–14.59)
Preterm	Preterm	46 (1.0)	26 (56.5)	36.19 (19.72–66.40)	38.24 (20.63–70.85)	21 (45.7)	37.88 (20.49–70.04)	51.90 (27.28–97.71)

CI, confidence interval; OR, odds ratio; PTB, preterm birth (<37 weeks’ gestation); sPTB, spontaneous preterm birth. * Values reflect the results of multivariable logistic regression analysis to determine the association between prior preterm delivery and the risk for preterm delivery in the third delivery. Models are adjusted for maternal age, chronic hypertension, hypertensive disorders of pregnancy (gestational hypertension and preeclampsia) and small for gestational age status (birthweight <10th centile) at the third delivery.

**Table 3:** The risk for spontaneous preterm birth in the third delivery stratified by history of spontaneous preterm births.

Study Subgroups			Risk for Spontaneous PTB (<37 Weeks) in 3rd Delivery			Risk for Spontaneous PTB (<34 Weeks) in 3rd Delivery		
1st Delivery	2nd Delivery	*N* (%)	Rate of PTB *n* (%)	Crude OR (95% C.I)	Adjusted OR * (95% C.I)	Rate of PTB *n* (%)	Crude OR (95% C.I)	Adjusted OR * (95% C.I)
Term	Term	4038 (90.3)	65 (1.6)	reference	reference	9 (0.2)	reference	reference
sPTD	Term	75 (1.7)	4 (5.3)	2.14 (0.76–5.94)	3.28 (1.15–9.33)	1 (1.3)	4.23 (0.55–32.59)	6.33 (0.79–50.74)
Term	sPTD	87 (1.9)	16 (18.4)	9.56 (5.37–17.02)	14.55 (7.98–26.53)	1 (1.1)	3.63 (0.47–27.91)	5.18 (0.65–41.37)
sPTD	sPTD	15 (0.3)	7 (46.7)	34.58 (12.32–97.04)	57.74 (20.21–164.95)	1 (6.7)	22.67 (2.79–184.28)	31.41 (3.71–265.79)

CI. confidence interval; OR, odds ratio; PTB. preterm birth; sPTB, spontaneous preterm birth. * Values reflect the results of multivariable logistic regression analysis to determine the association between prior spontaneous preterm birth and the risk for spontaneous preterm birth in the third delivery. Models are adjusted for maternal age, chronic hypertension, hypertensive disorders of pregnancy (gestational hypertension or preeclampsia) and small for gestational age (birthweight < 10th centile) status at the third delivery.

**Table 4:** The risk for spontaneous preterm birth in the third delivery stratified history of indicated preterm births.

Study Subgroups			Risk for Spontaneous PTB (<37 Weeks) in 3rd Delivery			Risk for Spontaneous PTB (<34 Weeks) in 3rd Delivery		
1st Delivery	2nd Delivery	*N* (%)	Rate of PTB *n* (%)	Crude OR (95% C.I)	Adjusted OR * (95% C.I)	Rate of PTB *n* (%)	Crude OR (95% C.I)	Adjusted OR * (95% C.I)
Term	Term	4038 (90.3)	65 (1.6)	reference	reference	9 (0.2)	reference	reference
iPTB	Term	146 (3.3)	7 (4.8)	1.93 (0.88–4.22)	3.03 (1.35–6.77)	1 (0.7)	2.12 (0.27–16.26)	3.31 (0.42–26.31)
Term	iPTB	80 (1.8)	4 (5.0)	1.99 (0.71–5.54)	3.26 (1.15–9.21)	0 (0)	-	-
iPTB	iPTB	15 (0.3)	5 (33.3)	19.39 (6.52–57.68)	32.91 (10.43–103.82)	1 (6.7)	22.67 (2.79–184.28)	43.87 (4.91–391.83)

CI, confidence interval; iPTB, indicated preterm birth <37 weeks’ gestation; OR, odds ratio; * Values reflect the results of multivariable logistic regression analysis to determine the association between prior indicated preterm birth and the risk for spontaneous preterm birth in the third delivery. Models are adjusted for maternal age, chronic hypertension, hypertensive disorders of pregnancy (gestational hypertension or preeclampsia) and small for gestational age status (birthweight < 10th centile) at the third delivery.
